# A lesson on induction of hypothermia and measurement of efficacy

**DOI:** 10.1186/s13054-014-0710-y

**Published:** 2014-12-22

**Authors:** Bridget A Harris, Peter JD Andrews

**Affiliations:** NHS Lothian and University of Edinburgh, Critical Care Unit, Western General Hospital, Edinburgh, EH4 2XU UK; Department of Anaesthesia and Critical Care, University of Edinburgh and NHS Lothian, Western General Hospital, Edinburgh, EH4 2XU UK

## Abstract

Brain injuries caused by stroke are common and costly in human and resource terms. The result of stroke is a cascade of molecular and physiological derangement, cell death, damage and inflammation in the brain. This, together with infection, if present, commonly results in patients having an increased temperature, which is associated with worse outcome. The usual clinical goal in stroke is therefore to reduce temperature to normal, or below normal (hypothermia) to reduce swelling if brain pressure is increased. However, research evidence does not yet conclusively show whether or not cooling patients after stroke improves their longer-term outcome (reduces death and disability). It is possible that complications of cooling outweigh the benefits. Cooling therapy may reduce damage and potentially improve outcome, and head cooling targets the site of injury and may have fewer side effects than systemic cooling, but the evidence base is unclear.

The recent study by Poli and colleagues [1] is part of a suite of iCOOL studies in ischaemic and haemorrhagic stroke, conducted at Heidelberg and linked to EuroHYP-1 (European multicentre, randomised, phase III clinical trial of therapeutic hypothermia plus best medical treatment versus best medical treatment alone for acute ischaemic stroke [2]). Altogether the studies tested four different methods of inducing hypothermia for speed of brain cooling, feasibility and safety. Rhinochill and the Sovika head and neck cooling device [1] and cold infusions compared with Rhinochill (Rhinochill, Wallisellen, Switzerland, EU) (iCOOL 1 NCT01573117 [1]), EMCOOLS Flex.Pads (EMCOOLS, Brucknerstrasse 6/7a, 1040 Vienna, Austria) (iCOOL 2 NCT01584167) and EMCOOLS Brain.Pad (EMCOOLS, Brucknerstrasse 6/7a, 1040 Vienna, Austria) (iCOOL 3 NCT01584180). The methods of inducing hypothermia that are now included in the protocol for EuroHYP-1 are cold infusion (20 ml/kg 4°C isotonic sodium chloride or Ringer’s lactate over 30 to 60 minutes) with optional use of EMCOOLs Brain.Pad [3].

There has been an ongoing quest for methods of therapeutic cooling that reduce temperature rapidly, are portable and easily instigated and/or have the least side effects. Cold infusion as a method of inducing cooling has most often been studied in cardiac arrest but is currently being used in clinical trials of therapeutic hypothermia in stroke (EuroHYP-1 [3]) and traumatic brain injury (Eurotherm3235Trial [4] and POLAR (NCT00987688)). The attraction is that it is a relatively low-tech, readily available, portable method, requiring only a means of keeping the infusion fluid at 4°C. Direct brain cooling has a long history but there are few randomised controlled trials and most are of low quality [5]. One of the attractions has been the assumption that direct brain cooling has fewer side effects than systemic cooling but this has not been established [5]. Various methods of nasopharyngeal cooling have been reported in the literature [5], including Rhinochill and nasal [6] and pharyngeal balloons [7]. Rhinochill has been studied most (for example, [8]) and there is very limited human data on the pharyngeal balloon device [7] or the pharyngeal cooling system of Takeda. Springborg and colleagues [6] report the use of QuickCool nasal balloons in a mixed group of hyperthermic brain-injured patients. Temperature was measured in the oesophagus, bladder and (in some patients) intracranially. The goal of normothermia was not reliably achieved. As with Poli and colleagues [1], this research raises questions about bladder temperature as a proxy for intracranial temperature.

Perfluorocarbons are costly and their use in Rhinochill has been questioned on environmental grounds [9]. Although in the overall context of medical interventions Rhinochill may not have major environmental impact, this nevertheless warrants consideration. Use of Rhinochill requires patients to be intubated and is contraindicated with base of skull fracture, which limits its use in traumatic brain injury.

Recently another method of nasopharyngeal cooling has been reported by Fontaine and colleagues [10], with experimental evidence and a human case report. This method uses adiabatic expansion of gas pressure; 1 L compressed carbon dioxide delivered via a nasal cannula. Temperature is reduced because as the gas expands the pressure reduction transfers energy as work (very rapidly) and not as heat, although in practice there is some heat transfer as insulation is not perfect. Carbon dioxide is of course also a greenhouse gas and using it in this way requires patients to be intubated. Compressed air and oxygen were tested experimentally as alternatives and found to remove considerably less heat than carbon dioxide, but *in vivo* temperature differences were not significant. In their study, Poli and colleagues [1] show very nicely how different sites of temperature measurement reflect temperature change differently using intravenous and nasopharyngeal cooling. Where intracranial temperature is the key temperature of interest, as it arguably is in stroke and traumatic brain injury, their data show the importance of measuring this. As yet, however, there does not seem to be much appetite for targeting therapeutic cooling to intracranial temperature - invasive measurement is not clinically warranted in less severe stroke and traumatic brain injury - and core body temperature is the usual feedback parameter. In this case Poli and colleagues’ data [1] strongly suggest oesophageal temperature is the best proxy for intracranial temperature. One difficulty with this site of measurement is that with longer-term cooling, if drugs are given nasogastrically, this will affect the temperature readings [11]. Another is having to site both a nasogastric tube and an oesophageal temperature probe with attendant increased risk of sinusitis and abrasions. There have been moves to produce a nasogastric tube suitable for feeding/drug instillation, aspiration and temperature measurement but to our knowledge such a potentially useful device is not yet in commercial production.

The authors are to be congratulated on their comprehensive measurement of temperature reduction efficacy and clear presentation of data measured at multiple sites. The challenge is to move from evidence of efficacy (temperature reduction) to evidence of effectiveness and improved patient outcomes.
